# Test–Retest Data for Radiomics Feature Stability Analysis: Generalizable or Study-Specific?

**DOI:** 10.18383/j.tom.2016.00208

**Published:** 2016-12

**Authors:** Janna E. van Timmeren, Ralph T.H. Leijenaar, Wouter van Elmpt, Jiazhou Wang, Zhen Zhang, André Dekker, Philippe Lambin

**Affiliations:** 1 Department of Radiation Oncology (MAASTRO), GROW-School for Oncology and Developmental Biology, Maastricht University Medical Centre (MUMC), Maastricht, The Netherlands;; 2 Department of Radiation Oncology, Fudan University Shanghai Cancer Center, China; and; 3 Department of Oncology, Shanghai Medical College, Fudan University, China

**Keywords:** radiomics, test–retest, computed tomography

## Abstract

Radiomics is an objective method for extracting quantitative information from medical images. However, in radiomics, standardization, overfitting, and generalization are major challenges to be overcome. Test–retest experiments can be used to select robust radiomic features that have minimal variation. Currently, it is unknown whether they should be identified for each disease (disease specific) or are only imaging device-specific (computed tomography [CT]-specific). Here, we performed a test–retest analysis on CT scans of 40 patients with rectal cancer in a clinical setting. Correlation between radiomic features was assessed using the concordance correlation coefficient (CCC). In total, only 9/542 features have a CCC > 0.85. Furthermore, results were compared with the test–retest results on CT scans of 27 patients with lung cancer with a 15-minute interval. Results show that 446/542 features have a higher CCC for the test–retest analysis of the data set of patients with lung cancer than for patients with rectal cancer. The importance of controlling factors such as scanners, imaging protocol, reconstruction methods, and time points in a radiomics analysis is shown. Moreover, the results imply that test–retest analyses should be performed before each radiomics study. More research is required to independently evaluate the effect of each factor.

## Introduction

Radiomics is the procedure of extracting features from medical images taken in clinical practice, for example, computed tomography (CT), positron emission tomography or magnetic resonance imaging (1–7). It is the imaging features' quantitative properties that make radiomics an objective method to quantify the phenotype of the tumor. With radiomics, numerous features are extracted from each image. Therefore, one of the main pitfalls of radiomics is the risk of overfitting. This can be solved, in part, by following a strict policy of feature reduction, for example, with test–retest and multiple delineation studies (8, 9), to select only those robust features that provide repeatable and reproducible measurements. These studies are usually performed on imaging data sets following a fixed acquisition and reconstruction protocol and using the same methodology for feature extraction.

However, imaging features have been shown to be influenced by multiple factors, including the type of scanner, imaging settings, reconstruction parameters, delineation of the tumor, and the mathematics of extracting features. Several studies assessed the influence of different scanners and reconstruction settings (10–15). In addition, the effect of image discretization, for example, standardized uptake value in positron emission tomography radiomics, is a necessary step before feature extraction that requires investigation (16). Thus, it is evident that standardization is required to generalize the use of radiomics. Thus, a key and unanswered question is whether results from test–retest studies can be generalized or if these also depend on one or more of the same factors that affect imaging features.

Previously, the repeatability of radiomic features in CT imaging was tested in a “coffee-break” test–retest data set of patients with lung cancer, in which 2 scans where made on the same scanner with a 15-minute interval [RIDER data set (4, 17)] (4). In such a test, one assumes that the tumor has not changed and, therefore, that the radiomic feature values should be repeatable. However, it is unknown if robust features in one tumor site are the same as those in another tumor site. Moreover, it is unknown if radiomic features are also robust in a more clinical test–retest setting, in which the time between scans is in the order of days and different scanners and/or scanner settings are used. Because of data inhomogeneity in the clinical test–retest setting, we hypothesized that test–retest results of the coffee-break scenario are not generalizable to the clinical scenario. Moreover, we expected that different features may be robust at one tumor site compared with those at another tumor site. To test this hypothesis, we examined the robustness of radiomic features obtained from CT scans of patients with rectal cancer in a clinical scenario (18) and compared this to the robustness of radiomic features for a CT lung cancer data set in a coffee-break scenario. Moreover, the role of features' correlation with volume on the robustness of radiomic features was assessed to investigate this potential surrogate for feature robustness.

## Methodology

A clinical data set of 40 patients with rectal cancer was included in this study. For each patient, 2 CT scans were taken before treatment using Brilliance CT Big Bore (Philips Healthcare, Cleveland, OH). The RIDER data set of patients with lung cancer was included in the study as a second test–retest data set, described elsewhere (4, 17, 19–21). To avoid discrepancies between methods of analysis, results of the previous study were not reused but obtained again. In total, 27 patients of the RIDER data set were included in the final analysis (data of 5 patients could not be retrieved or had to be excluded because of technical problems). CT parameters are summarized in Table 1.

**Table 1.** CT Scan Parameters for Both Data sets

Parameters	Rectum Data Set	RIDER Data Set
Manufacturer	Philips Healthcare	GE Healthcare
Acquisition type	Helical	Helical
Tube voltage	120 kVp	120 kVp
Tube current	250 or 350 mAs	Range 165–549 mAs
Slice thickness	5 mm	1.25 mm
Pixel spacing	Range 0.98–1.25 mm	Range 0.51–0.91 mm
Pixels	512 × 512	512 × 512

We investigated the test–retest stability on both data sets of a total of 542 radiomic features, divided into the following 4 groups: (1) Tumor intensity (n = 15), (2) shape (n = 11), (3) texture (n = 44), and (4) wavelet (n = 472). Mathematical descriptions of all features are published elsewhere (4).

### Statistical Analysis

The concordance correlation coefficient (CCC) was used to examine agreement between radiomic features derived from the test–retest scan (22). CCCs were calculated for the RIDER data set and the rectum data set and each feature group was compared. For all features in the rectum data set, we also assessed the correlation with volume using a simple linear regression and R^2^ as correlation parameter. Statistical analysis was performed using the package *psych* in R (version 3.2.3).

## Results

**Figure 1.**
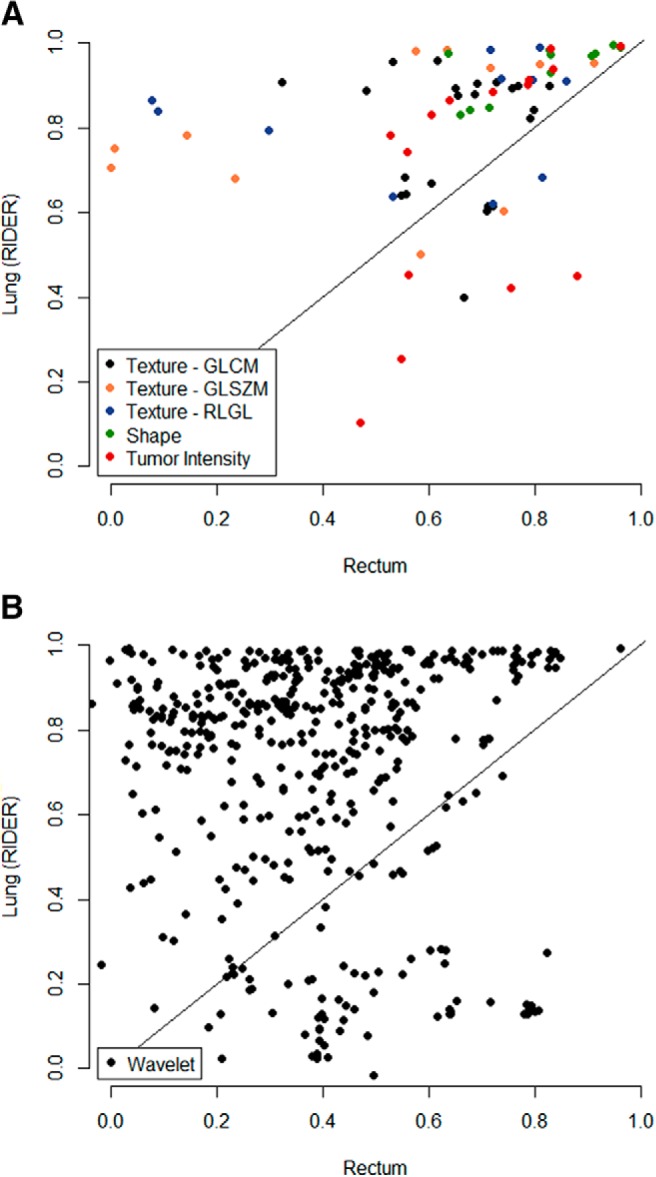
Comparison between stability of radiomic features derived from the lung cancer data set (RIDER) and the rectal cancer data set, with feature groups “Texture,” “Shape,” and “Tumor Intensity” (A) and “Wavelet” (B). Gray-level co-occurrence matrix (GLCM), gray-level size zone matrix (GLSZM), and run-length gray level (RLGL).

For each feature group, CCC values between the RIDER data set and the clinical data set were compared. Results are shown in Figure 1. This analysis was also performed after resampling all data into images with an isotropic voxel size of 3 mm before feature extraction. These results are shown in Supplemental Figure 1.

In total, for 446/542 features (82.3%), the data points are on the left side of the diagonal, meaning that they have a higher CCC in the RIDER data set than in the clinical data set. This is 36/44 features (81.8%) for the “Texture” group, 11/11 features (100%) for “Shape” group, 10/15 features (66.7%) for “Tumor Intensity” group, and 389/472 features (82.4%) for “Wavelet” group.

Shape features were the most reproducible (100% CCC > 0.6 for both sets), followed by the gray-level co-occurrence matrix features of the “Texture” group (91% and 68% CCC > 0.6 for the RIDER set and the clinical set, respectively). Wavelet features seemed to be the least reproducible in the clinical setting. When using a cutoff CCC of 0.85, only 9 features were reproducible in the clinical scenario-derived rectal cancer test–retest set, whereas 234 features were reproducible in the coffee-break lung cancer test–retest set; 8 of these feature overlapped.

Considering the 100 most stable features of the RIDER data set (range CCC = 0.951–0.995), 36 of those features were also in the 100 most stable features of the clinical data set, which is not likely to be found because of chance (*P* < .0001). The overlapping features included 7/11 “Shape” features (63.6%), 1/15 “Tumor intensity” features (6.7%), 2/44 “Texture” features (4.6%), and 26/472 “Wavelet” features (5.5%). The 100 most stable features of both data sets are displayed in Supplemental Table 1.

**Figure 2.**
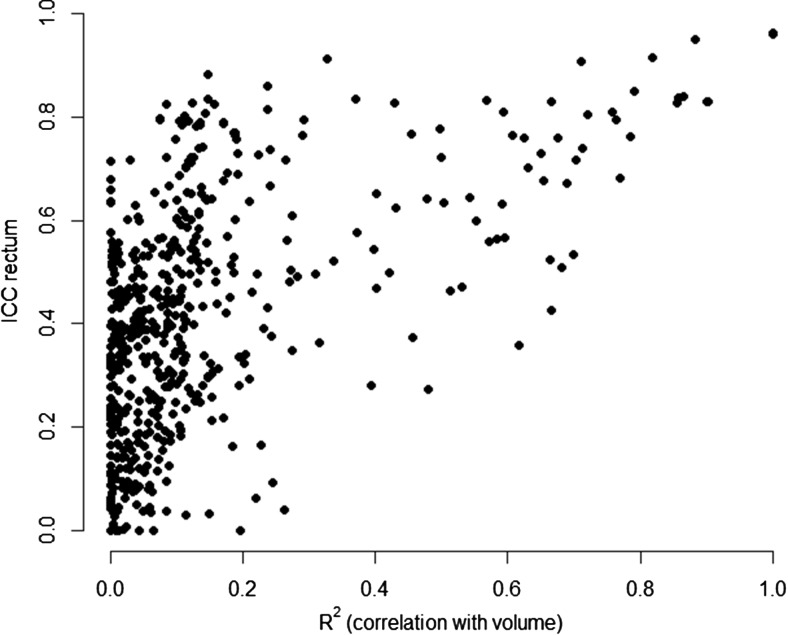
Robustness of radiomic features in the rectum data set (y-axis) versus the features' correlation with volume (x-axis).

For all features of the clinical rectum data set, we assessed the correlation with volume using the coefficient of determination (R^2^) of a simple linear regression. Features extracted from the test scan were used for this analysis. Results are shown in Figure 2. The y-axis represents the robustness of all features, and the x-axis represents the R^2^ of the correlation with volume.

In total, 4 features had an R^2^ value >0.9, meaning a high correlation with volume. These were “Tumor intensity–Total energy” (R^2^ = 1.0), “Wavelet–LLL Tumor intensity [L: Low-pass (4)]–Total energy” (R^2^ = 1.0), “Tumor intensity–Energy” (R^2^ = 0.90), and “Wavelet–LLL Tumor intensity energy” (R^2^ = 0.90). Features were generally more robust with an increase in correlation with volume.

## Discussion

In comparison to the RIDER data set with the “coffee-break” scenario, features were much less stable in a more clinical scenario of repeated imaging. However, the results show that a subset of stable features derived from the RIDER data set is also stable in the clinical scenario. There is particularly high correspondence between the “Shape” features, whereas correspondence between “Wavelet” features is low. The low correspondence between the clinical RIDER data sets can be because of many factors, including the disease site, time between scans, and the CT settings. Slice thickness is one of the factors that differs considerably between the CT scans of patients with rectal cancer (5 mm) and those with lung cancer (1.25 mm). However, resampling the data into isotropic voxels of 3 mm before feature extraction did not substantially change the results. Slightly more features were robust after resampling, but the difference in stability between both data sets is similar. The stability of radiomic features may be disease-specific and a function of the time between scans, but we are not able to eliminate the effect of protocol differences between both data sets. If we could show that numerous features are robust in a clinical scenario and that these features correspond to the robust features found by a “real” test–retest analysis (ie, with a very short time interval), this could have implied that one extensive test–retest study could provide a set of stable radiomic features that could be used in any further radiomics analysis. However, as this appeared to be not the case, we emphasize, in this study, the importance of a proper test–retest study in each scenario with a tight control on influencing parameters and further investigation of the influence of factors such as hardware, scan acquisition and reconstruction settings, tumor delineation, and the mathematics of extracting features.

Volume was one of the robust features in the clinical test–retest analysis (CCC = 0.96). Of the 100 most stable features in the rectum data set, 4 other features highly correlated with volume. Moreover, when the correlation with volume was increasing, features were also more robust. This could partly explain the stability of these features. In total, only a small portion of radiomic features, 9/542, was robust in the clinical test–retest analysis (CCC > 0.85), which suggested that differences between the 2 CT scans influence the stability of the radiomic features. Various causes may explain this. For example, for a subset of 17 patients, a different tube current was used for the test–retest scan—250 mAs and 350 mAs or vice versa—leading to different noise properties in both scans. Another factor that could have reduced the robustness of radiomic features is the voxel resolution because of different reconstruction diameters, which was not constant over all CT scans. Moreover, the number of days between the test–retest scan ranged from 5 to 19 (median 8), which is not comparable to the 15-minute interval in the RIDER data set. It cannot be excluded that in this time period, the tumor changes sub-clinically and that this change is detected by radiomics. Therefore, if this data set is used for test–retest analysis as shown in this study, it would mean that we discard features that are actually informative. If such rapid changes occur in an untreated tumor, future protocols should closely control the time of the CT (eg, the time between CT and treatment) so that the scan is taken at the time when it is the most informative. When prognostic information is derived from image features in a radiomics study, one should be aware of changes in a tumor, and it is advisable to avoid using features that are not robust in a test–retest study, in which the time interval between scans is large. Future studies, where the predictive performance for the outcome of interest is investigated, of images taken at different time points before treatment are necessary to address these considerations.

Several factors could have reduced the robustness of the radiomic features. In this study, the radiomics methodology is controlled, but the hardware, scan acquisition and reconstruction settings, disease site, and scan time interval are different. This study shows that test–retest results are not generalizable, and there is a dependency on one or a combination of these factors. Ideally, one should alter only one of these factors at a time for testing the influence. However, the number of patients in a test–retest analysis is usually low. Although in this study we had numerous patients for a test–retest study (n = 40), the data set is very small to be able to analyze subsets to test these effects. Phantom measurements could play an important role in accurately assessing the potential influence of differences in scanners, reconstruction methods, and imaging settings on radiomic features and may allow for a calibration of the feature values. To conclude, we emphasize that it is important to tightly control all aforementioned factors in a radiomics study. Nevertheless, to minimize the risk of using unstable and unreproducible features in a radiomics analysis, it is advisable to perform treatment site-specific and time-, scanner-, and imaging protocol-controlled test–retest analyses.

### Supplemental Materials

Supplemental Figure 1:

Supplemental Table 1:
