# Supplementary material for: Test–Retest Data for Radiomics Feature Stability Analysis: Generalizable or Study-Specific?
Source: Tomography. 2016 Dec;2(4):361–5. doi: 10.18383/j.tom.2016.00208 (PMC6037932; doi:10.18383/j.tom.2016.00208)
Supplement: Supplemental Figure 1: [file tom-00208-16-s001.pdf]

Supplemental Data

Prior to feature extraction, all data were resampled into images with a voxel size of 3 x 3 x 3 mm. Results are shown in Figure 1. In total, for 530 out of 542 (97.8%) of the features the data points are on the left side of the diagonal, meaning that they have a higher CCC in the RIDER dataset than in the clinical dataset.

When using the cut-off of CCC 0.85, 17 features where reproducible in the clinical scenario derived rectal cancer test-retest set while 264 features where reproducible in the coffee-break lung cancer test-retest set. 16 of these feature overlapped. Slightly more features were robust after resampling for both the rectal cancer dataset (17 vs. 9) and the lung cancer dataset (264 vs. 234).

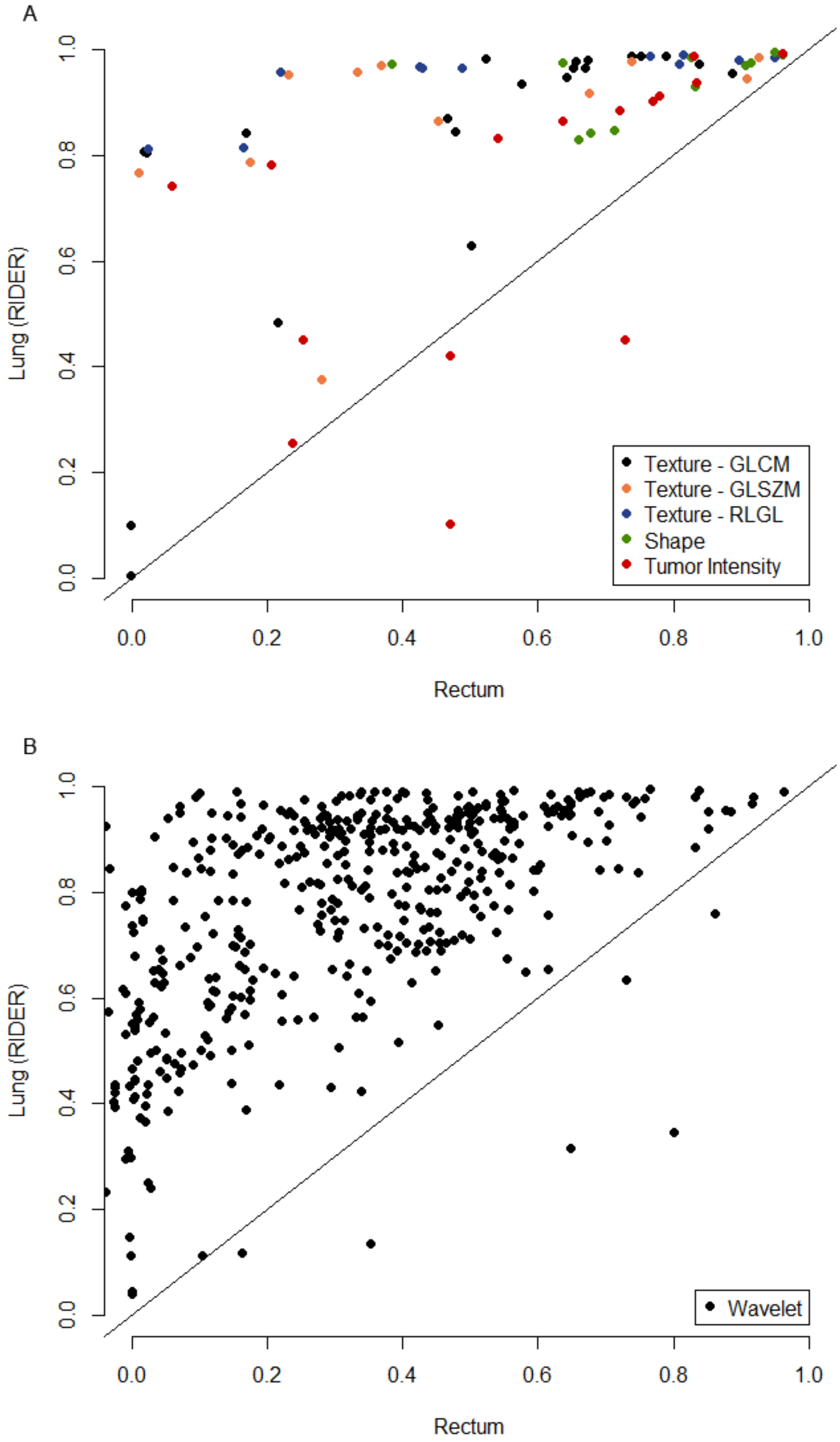

Figure 1: Comparison between stability of radiomic features derived from the lung cancer dataset (RIDER) and the rectal cancer dataset, with in panel ***A)*** *Feature groups 'Texture', 'Shape' and 'Tumor Intensity' and in* ***B)*** *'Wavelet'. Images were resampled into isotropic voxels of 3 mm prior to feature extraction. GLCM: grey-level co-occurrence matrix, GLSZM: grey-level size zone matrix and RLGL: run-length grey-level.*
