# Supplementary material for: Test–Retest Data for Radiomics Feature Stability Analysis: Generalizable or Study-Specific?
Source: Tomography. 2016 Dec;2(4):361–5. doi: 10.18383/j.tom.2016.00208 (PMC6037932; doi:10.18383/j.tom.2016.00208)
Supplement: Supplemental Table 1: [file tom-00208-16-s002.pdf]

Supplemental Data

Table 1: 100 most robust features for both datasets: rectal cancer (left two columns) and lung cancer (right two columns). Common features are indicated bold. Note that feature group 'Tumor intensity' is shortened to 'Stats'.

Rectal cancer		Lung cancer	
Feature name	CCC	Feature name	CCC
**Wavelet_LLL_stats_totalenergy**	**0.962**	**Shape_compactness**	**0.995**
**Stats_totalenergy**	**0.961**	**Stats_totalenergy**	**0.992**
**Shape_volume**	**0.960**	Wavelet_HLH_glcm_infoCorr1	0.992
**Shape_compactness**	**0.948**	**Wavelet_LLL_stats_totalenergy**	**0.992**
**Shape_surface**	**0.913**	**Wavelet_LHH_glszm_sizeZoneVariability**	**0.992**
GLSZM_highIntensityLarteAreaEmp	0.911	**RLGL_grayLevelNonuniformity**	**0.990**
**Shape_maxDiameter2D3**	**0.905**	**Shape_volume**	**0.990**
Stats_kurtosis	0.879	Wavelet_LLH_rlgl_runLengthNonuniformity	0.988
RLGL_longRunEmphasis	0.856	Wavelet_HLL_glcm_infoCorr1	0.988
**Wavelet_HHH_rlgl_grayLevelNonuniformity**	**0.846**	Wavelet_HHH_glcm_infoCorr1	0.988
**Wavelet_LHL_rlgl_grayLevelNonuniformity**	**0.836**	Wavelet_LHL_glcm_infoCorr1	0.988
Wavelet_LLH_rlgl_grayLevelNonuniformity	0.835	Wavelet_HLH_rlgl_runLengthNonuniformity	0.988
Stats_median	0.832	Wavelet_LLL_glszm_intensityVariability	0.987
**Wavelet_LLL_glszm_highIntensityLargeAreaEmp**	**0.832**	**Wavelet_LHH_rlgl_runLengthNonuniformity**	**0.987**
Shape_maxDiameter2D2	0.828	**Wavelet_HLH_glszm_intensityVariability**	**0.987**
**Stats_energy**	**0.827**	Wavelet_HLL_rlgl_runLengthNonuniformity	0.987
**Wavelet_LLL_stats_energy**	**0.827**	**Stats_energy**	**0.987**
**Shape_maxDiameter3D**	**0.826**	Wavelet_HHL_rlgl_runLengthNonuniformity	0.987
GLCM_inverseVar	0.824	**Wavelet_LLL_stats_energy**	**0.987**
**Shape_surfVolRatio**	**0.824**	Wavelet_LHL_glszm_intensityVariability	0.987
**Wavelet_HLL_rlgl_grayLevelNonuniformity**	**0.824**	Wavelet_HHL_glszm_intensityVariability	0.987
Wavelet_LLL_stats_median	0.822	**Wavelet_HHH_rlgl_runLengthNonuniformity**	**0.987**
Wavelet_LHH_glcm_clusProm	0.820	Wavelet_LLH_glszm_intensityVariability	0.986
RLGL_longRunHighGrayLevEmpha	0.810	**Wavelet_LHH_glszm_intensityVariability**	**0.986**
**RLGL_grayLevelNonuniformity**	**0.807**	Wavelet_HHH_glcm_infoCorr2	0.986
GLSZM_largeAreaEmphasis	0.805	**Wavelet_LHL_rlgl_runLengthNonuniformity**	**0.985**
Wavelet_HLH_glcm_sumVar	0.803	Wavelet_HLH_glcm_infoCorr2	0.985
**Wavelet_LLL_rlgl_grayLevelNonuniformity**	**0.801**	**RLGL_runLengthNonuniformity**	**0.985**
Wavelet_HHL_glszm_highIntensityEmphasis	0.797	Wavelet_HHL_glcm_infoCorr2	0.985
GLCM_energy	0.794	Wavelet_LLL_rlgl_runLengthNonuniformity	0.985
RLGL_runPercentage	0.791	Wavelet_LHL_glcm_infoCorr2	0.985
**Wavelet_HLL_stats_median**	**0.790**	**Wavelet_LHL_rlgl_grayLevelNonuniformity**	**0.984**
**Wavelet_HLH_rlgl_grayLevelNonuniformity**	**0.790**	**Shape_surfVolRatio**	**0.984**
Wavelet_HHL_glcm_sumVar	0.789	Wavelet_HLL_glszm_intensityVariability	0.983
GLCM_maxProb	0.788	**Wavelet_LLL_rlgl_grayLevelNonuniformity**	**0.982**
Wavelet_HHL_rlgl_highGrayLevelRunEmphasis	0.787	GLSZM_intensityVariability	0.982
Wavelet_HLH_glcm_autocorr	0.784	Wavelet_LHL_glszm_highIntensityLarteAreaEmp	0.981
Stats_rms	0.784	Wavelet_HLL_stats_kurtosis	0.981

Stats_mean	0.783
Wavelet_HLH_glcm_sumSquares	0.782
Wavelet_HHL_glcm_autocorr	0.781
Wavelet_HLH_rlgl_highGrayLevelRunEmphasis	0.781
Wavelet_HHL_glcm_sumSquares	0.780
Wavelet_HLH_glszm_highIntensityEmphasis	0.777
**Wavelet_HHL_glszm_highIntensityLargeAreaEmp**	**0.772**
Wavelet_LLL_stats_rms	0.765
GLCM_homogeneity1	0.764
**Wavelet_LHH_glszm_sizeZoneVariability**	**0.762**
**Wavelet_HHH_stats_energy**	**0.761**
Wavelet_LLL_stats_mean	0.761
**Wavelet_LHL_stats_median**	**0.759**
**Wavelet_LHH_rlgl_grayLevelNonuniformity**	**0.758**
**Wavelet_HHH_stats_totalenergy**	**0.755**
Wavelet_HLL_glszm_highIntensityLarteAreaEmp	0.754
GLCM_homogeneity2	0.753
Stats_skewness	0.751
GLSZM_highIntensityEmphasis	0.737
Wavelet_LLL_glszm_highIntensityEmphasis	0.735
**Wavelet_HHH_rlgl_runLengthNonuniformity**	**0.734**
RLGL_shortRunEmphasis	0.731
**Wavelet_LHH_rlgl_runLengthNonuniformity**	**0.725**
GLCM_dissimilar	0.723
Wavelet_LLL_rlgl_longRunHighGrayLevEmpha	0.723
GLCM_sumVar	0.717
RLGL_highGrayLevelRunEmphasis	0.717
Stats_uniformity	0.717
**Wavelet_LHL_rlgl_runLengthNonuniformity**	**0.716**
GLSZM_zonePercentage	0.712
**RLGL_runLengthNonuniformity**	**0.712**
Wavelet_LLL_glcm_clusProm	0.712
Wavelet_LLL_rlgl_highGrayLevelRunEmphasis	0.710
Shape_spherDisprop	0.709
Wavelet_LLL_glcm_sumVar	0.708
GLCM_autocorr	0.706
GLCM_sumSquares	0.706
Wavelet_LLL_glcm_autocorr	0.698
Wavelet_LLL_glcm_sumSquares	0.697
**Wavelet_LHH_stats_energy**	**0.696**
GLCM_diffEntro	0.686
Wavelet_LLL_glcm_correl1	0.684
GLCM_entrop2	0.682
**Wavelet_LHH_glszm_intensityVariability**	**0.676**
Shape_sphericity	0.673
Wavelet_HHH_glszm_intensityVariability	0.981
GLSZM_sizeZoneVariability	0.980
Wavelet_LHH_glcm_infoCorr1	0.980
Wavelet_HLL_glcm_infoCorr2	0.980
Wavelet_LHH_glcm_infoCorr2	0.979
**Wavelet_LLL_glszm_highIntensityLargeAreaEmp**	**0.979**
Wavelet_LLH_glcm_infoCorr2	0.978
Wavelet_LLL_glszm_zonePercentage	0.978
**Wavelet_LHH_stats_energy**	**0.977**
Wavelet_LLH_stats_energy	0.977
**Wavelet_HLL_stats_median**	**0.976**
**Shape_surface**	**0.975**
Shape_maxDiameter2D1	0.975
Wavelet_HLL_stats_mean	0.974
Wavelet_HHL_glszm_sizeZoneVariability	0.974
**Wavelet_LHH_rlgl_grayLevelNonuniformity**	**0.974**
**Shape_maxDiameter3D**	**0.972**
**Wavelet_HHH_stats_energy**	**0.972**
Wavelet_HLH_stats_energy	0.971
**Wavelet_HHL_rlgl_grayLevelNonuniformity**	**0.970**
**Wavelet_HHH_glszm_sizeZoneVariability**	**0.970**
Wavelet_HLH_glszm_sizeZoneVariability	0.970
Wavelet_HHL_stats_energy	0.970
**Shape_maxDiameter2D3**	**0.970**
**Wavelet_HHH_rlgl_grayLevelNonuniformity**	**0.969**
Wavelet_HHL_stats_totalenergy	0.968
Wavelet_LHL_glszm_sizeZoneVariability	0.968
Wavelet_LHL_glszm_zonePercentage	0.968
Wavelet_LHL_glszm_largeAreaEmphasis	0.967
Wavelet_LLH_glcm_infoCorr1	0.967
Wavelet_LLL_glszm_sizeZoneVariability	0.966
**Wavelet_HLL_rlgl_grayLevelNonuniformity**	**0.965**
Wavelet_LLL_glszm_largeAreaEmphasis	0.964
**Wavelet_LHH_stats_totalenergy**	**0.964**
Wavelet_HLL_stats_totalenergy	0.963
Wavelet_HLL_glszm_sizeZoneVariability	0.962
Wavelet_LLL_glcm_inverseVar	0.962
Wavelet_HHL_glcm_infoCorr1	0.962
Wavelet_HHL_glcm_invDiffnorm	0.961
Wavelet_HLL_glcm_invDiffnorm	0.961
Wavelet_LHL_stats_totalenergy	0.961
Wavelet_HLL_stats_energy	0.961
**Wavelet_HHL_glszm_highIntensityLargeAreaEmp**	**0.961**
Wavelet_LLH_stats_totalenergy	0.960
**Wavelet_LLL_glcm_infoCorr1**	**0.959**

Wavelet_LLL_rlgl_runPercentage	0.672	Wavelet_LHL_glcm_invDiffnorm	0.959
**Wavelet_LHH_stats_totalenergy**	**0.671**	Wavelet_LHL_glcm_invDiffmomnor	0.958
**Wavelet_HHL_rlgl_grayLevelNonuniformity**	**0.666**	GLCM_infoCorr1	0.958
GLCM_clusProm	0.662	**Wavelet_LHL_stats_median**	**0.957**
**Wavelet_LLH_stats_median**	**0.661**	Wavelet_LLH_glszm_sizeZoneVariability	0.956
Wavelet_LLL_stats_kurtosis	0.659	Wavelet_LLL_glcm_infoCorr2	0.955
Shape_compactness2	0.655	**Wavelet_HLH_rlgl_grayLevelNonuniformity**	**0.954**
GLCM_sumEntro	0.649	Wavelet_LLL_glcm_homogeneity2	0.954
Wavelet_HHL_glszm_highIntensitySmallAreaEmp	0.648	Wavelet_LLL_glcm_homogeneity1	0.954
Wavelet_LLL_glcm_contrast	0.646	**Wavelet_LLH_stats_median**	**0.954**
**Wavelet_LLL_glcm_infoCorr1**	**0.645**	Wavelet_LHL_stats_mean	0.953
GLCM_contrast	0.645	GLCM_infoCorr2	0.953
**Wavelet_HLH_glszm_intensityVariability**	**0.639**	Wavelet_LHL_stats_md	0.953
Wavelet_LHH_rlgl_highGrayLevelRunEmphasis	0.636	Wavelet_LLL_rlgl_shortRunEmphasis	0.953
Wavelet_LHH_glcm_sumVar	0.635	Wavelet_LHL_stats_energy	0.952
**Wavelet_HHH_glszm_sizeZoneVariability**	**0.635**	Wavelet_LLL_stats_uniformity	0.952
Wavelet_LHH_glcm_sumSquares	0.635	**Wavelet_HHH_stats_totalenergy**	**0.951**
